# Depot-Specific Cardiorenal Adipose Remodeling with SGLT2i in Chronic Kidney Disease

**DOI:** 10.3390/jcm15103641

**Published:** 2026-05-09

**Authors:** Ana Checa-Ros, Óscar Arias, Owahabanun-Joshua Okojie, Pilar Salvador, Luis D’Marco

**Affiliations:** 1 Grupo de Investigación en Enfermedades Cardiorrenales y Metabólicas, Departamento de Medicina y Cirugía, Facultad de Ciencias de la Salud, Universidad Cardenal Herrera-CEU, CEU Universities, C/Santiago Ramón y Cajal s/n, Alfara del Patriarca, 46115 Valencia, Spain; ana.checaros@uchceu.es (A.C.-R.); oscar.ariasmutis@uchceu.es (Ó.A.); joshua.okojie1@alumnos.uchceu.es (O.-J.O.); 2 Hospital Vithas Consuelo, 46007 Valencia, Spain; salvadormp@vithas.es

**Keywords:** adiposopathy, chronic kidney disease, epicardial adipose tissue, perirenal adipose tissue, sodium–glucose cotransporter 2 inhibitors

## Abstract

**Background and hypothesis:** Sodium–glucose cotransporter 2 inhibitors (SGLT2i) provide consistent cardiorenal benefits; however, tissue-level mechanisms remain insufficiently characterized. We investigated whether SGLT2i were associated with longitudinal remodeling of organ-specific adipose depots in patients with chronic kidney disease (CKD). **Methods:** In this observational study cohort (ADIPO-CKD; NCT07309094), adults with CKD stages 1–4 underwent clinical, biochemical and ultrasound imaging assessment at baseline (T0) and 8-month follow-up (T8). Thus, epicardial (EAT) and perirenal adipose tissue (PRAT) thickness were measured. Changes over time between patients under SGLT2i treatment and those without (Non-SGLT2i) were assessed using repeated-measures ANOVA and multivariable linear regression models adjusted for age, sex, baseline estimated glomerular filtration rate (eGFR), diabetes status, concomitant glucagon-like peptide 1 (GLP-1) receptor agonist therapy, body mass index (BMI) and visceral fat area (VFA) changes. **Results:** Among 189 CKD patients (50 SGLT2i and 139 non-SGLT2i), SGLT2i therapy was associated with significant reductions in PRAT (1.28 ± 0.70 to 0.91 ± 0.61 cm; ΔPRAT −0.37 cm; *p* < 0.002) and EAT (0.57 ± 0.27 to 0.36 ± 0.14 cm; ΔEAT −0.21 cm; *p* < 0.012), whereas no significant changes were observed in the Non-SGLT2i group. In multivariable models, SGLT2i exposure remained independently associated with ΔPRAT (β = 0.447; 95% CI 0.211–0.682; *p* < 0.001; R^2^ = 0.371) and ΔEAT (β = 0.061; 95% CI 0.009–0.113; *p* < 0.021; R^2^ = 0.053), including adjustment for changes in BMI and VFA. These findings were accompanied by trends toward improvement in renal function and systemic inflammation biomarkers in the SGLT2i group, although these changes did not reach statistical significance. In a secondary analysis, dapagliflozin was significantly associated with PRAT reduction, whereas a significant association was found between empagliflozin and EAT decrease. **Conclusions:** In CKD stages 1–4, SGLT2i use was independently associated with reductions in EAT and PRAT. These findings support a potential link between organ-specific adipose tissue and cardiorenal disease; however, given the observational design, these results should be interpreted as associative and hypothesis-generating. Dedicated mechanistic and adequately powered studies are warranted to determine their clinical relevance.

## 1. Introduction

Ectopic adipose tissue surrounding vital organs has emerged as an active contributor to cardiorenal and metabolic diseases rather than a passive fat reservoir [1]. In patients with chronic kidney disease (CKD), cardiovascular morbidity and mortality remain disproportionately high, and in addition to traditional risk factors, this ectopic tissue may incompletely explain this excess risk [2]. Increasing evidence suggests that beyond total adiposity, the anatomical distribution and functional phenotype of organ-specific fat depots may critically influence cardiorenal outcomes. Among these depots, epicardial adipose tissue (EAT) and perirenal adipose tissue (PRAT) are of particular interest due to their direct anatomical and functional proximity to the myocardium and renal parenchyma, respectively [3,4,5,6].

EAT shares coronary microcirculation and lacks a fascial barrier with the myocardium, facilitating paracrine and vasocrine signaling that may promote inflammation, myocardial remodeling, and adverse cardiovascular outcomes [3,7]. Similarly, PRAT envelops the kidney within Gerota’s fascia and maintains close vascular and neural connections with the renal cortex, potentially influencing intrarenal hemodynamics, sodium handling, and inflammatory pathways relevant to CKD progression [8,9,10].

Sodium–glucose cotransporter 2 inhibitors (SGLT2i) have consistently demonstrated cardiovascular and renal protection across diverse populations [11,12,13]. While several hemodynamic and metabolic mechanisms have been proposed, their potential impact on organ-specific adipose depots remains insufficiently characterized. EAT and PRAT represent anatomically distinct yet functionally interconnected depots capable of influencing myocardial and renal physiology through local paracrine signaling.

In this study, we sought to determine whether SGLT2 inhibition influences organ-specific adipose compartments and whether such modulation is associated with markers of cardiorenal risk. Consequently, this CKD cohort study evaluated the longitudinal impact of SGLT2i therapy on both EAT and PRAT thickness and remodeling, while also exploring whether these associations were consistent across different SGLT2i agents.

## 2. Materials and Methods

### 2.1. Design

This is a preliminary analysis from the ADIPO-CKD research project (NCT07309094), an ongoing observational and prospective real-life cohort study carried out on a hospital-based clinical sample with varied cardio-renal-metabolic spectrum, including patients with CKD and/or cardiovascular diseases (CVDs), type 2 diabetes mellitus (T2DM) and obesity. The purpose of the ADIPO-CKD project is to establish a link between adiposopathy and renal function markers via the changes observed in adiposopathy, inflammation and renal function parameters throughout follow-up treatment with the antidiabetic medications, such as glucagon-like peptide-1 receptor agonists (GLP-1RAs) and SGLT2is.

For this cohort, we assessed the changes in PRAT, EAT, adiposopathy, inflammation and renal function biomarkers throughout the follow-up of patients with CKD stages 1–4, with or without T2DM and comparing those receiving SGLT2i with those not receiving this medication (Non-SGLT2i group).

### 2.2. Patient Characteristics

Patients were recruited from the Nephrology and Internal Medicine Departments at the Virgen del Consuelo Hospital (Valencia, Spain), between July 2023 and January 2025. They were invited to participate in the study if they met all the following inclusion criteria: (a) ≥18 years of age, due to those of a younger age not being able to fully understand and give an informed consent; (b) diagnosed with CKD in stages G1, G2, G3a, G3b, and G4, not candidate for dialysis, as SGLT2i is currently contraindicated for use in patients who are in end-stage kidney disease (ESKD) or in dialysis [14]; (c) had T2DM and/or CVD, since SGLT2is are currently used in treatments of these diseases/conditions [15,16]; and (d) were willing to participate in the study and sign informed consent. Of note, patients who were candidates for SGLT2i treatment were assigned to the SGLT2i group following the Spanish Society of Nephrology guidelines [17], whereas non-candidates were included in the Non-SGLT2i group. The CKD staging was established according to the estimated glomerular filtration rate (eGFR) as per the current KDIGO guidelines (G1: eGFR ≥ 90 mL/min/1.73 m^2^; G2: 60–89 mL/min/1.73 m^2^; G3a: 45–59 mL/min/1.73 m^2^; G3b: 30–44 mL/min/1.73 m^2^; G4: 15–29 mL/min/1.73 m^2^) [18]. Age < 18 years, pregnancy (SGLT2i is contraindicated in pregnancy) [19], CKD in stage G5 or G4 candidate for dialysis, neuropsychiatric diseases preventing the patient from understanding the benefits/risks associated with the project, refusal to participate and/or consent revocation were considered as exclusion criteria.

Clinical, analytical, and imaging data collection was performed upon recruitment (T0) and after an average follow-up of 8 months (T8) in both cohorts. For the SGLT2i cohort, T0 represented the measure prior to treatment, whereas T8 was the measure after 8 months of treatment.

### 2.3. Outcomes

#### 2.3.1. Clinical Data Collection

Patient electronic medical records (EMRs) from the hospital institutional software (Vithas ONE v1.0) and study specific Case Report Forms (CRF) were used to collect the following demographic and clinical information: age; sex; height; cardiovascular risk factors (arterial hypertension, T2DM, smoking, obesity, hypercholesterolemia); CVD load based on personal history of stroke, myocardial infarct and peripheral vascular disease, and scored as 0 = none, 1 = personal history of CVD, 2 = personal history of 2 CVDs and 3 = personal history of 3 CVDs; other comorbidities; treatment history, including: angiotensin-converting enzyme inhibitors or ACE inhibitors, angiotensin receptor blockers or ARBs, calcium channel blockers or CCBs, diuretics, mineralocorticoid receptor antagonists or MRAs, beta-blockers, statins, fibrates, anti-hyperuricemia drugs, vitamin-D based treatments, insulin, metformin, dipeptidyl peptidase 4 inhibitors (DPP4i) and GLP-1RA.

Body composition analysis was performed through bioimpedance with the optimized body composition analyzer i35 (Mediana Co., Ltd., Flowery Branch, GA, USA).

#### 2.3.2. Analytical Data Collection

Blood and urine samples were collected from patients in both cohorts at T0 and T8 to measure renal and cardiometabolic function, as well as inflammation and adiposopathy markers, such as tumor necrosis factor-alpha (TNF-α), interleukin-6 (IL-6), C-reactive protein (CRP), ferritin and leptin, among other common parameters. Blood samples were obtained after 8 to 12 h of fasting and a 15-min resting period. Upon blood clot formation, they were centrifuged at 3500 rpm for 10 min to obtain the serum. First morning urine samples were collected from patients. Laboratory assays involved potentiometry and kinetic colorimetric/photometric analyses on an automated analyzer (ROCHE^®^ model c311; Roche Diagnostics, Barcelona, Spain). After processing, samples were stored at a temperature between −16°C and −28°C in the Laboratory Unit at the Virgen del Consuelo Hospital and then transported to the laboratories at CEU Cardenal Herrera University for further storage in the biobank (at −80 °C).

#### 2.3.3. Imaging Data Collection

The PRAT was assessed with the patient being placed in a supine position. A bilateral renal evaluation was performed, and the kidneys were visualized in the anteroposterior, transverse and longitudinal directions. The PRAT was measured in the distal third between the cortex and hepatic border and/or the spleen. The liver/spleen was visualized in a longitudinal view, and the echogenicity of the liver/spleen parenchyma was assessed and compared to the right and left kidney, respectively.

To measure the EAT, the patient was placed in a left lateral decubitus position in order to obtain a parasternal long-axis view of the heart. The EAT thickness was then measured between the outer wall of the myocardium and the visceral layer of the pericardium at the right ventricle.

The Mindray^®^ TEAir e5M ultrasonography (Mindray^®^, Shenzhen, China) was used to perform measurements (in centimeters) using the abdominal B-mode ultrasound technique.

All the images were then stored in DICOM format for further processing by an expert radiologist blinded to the patients’ data. Intra- and inter-observer variability were not systematically assessed in this study and therefore could not be reported, which represents an additional limitation regarding measurement reproducibility.

### 2.4. Statistics

Continuous variables were presented as mean ± standard deviation (SD) or median and interquartile range (IQR), according to distribution assessed by the Shapiro–Wilk test. Categorical variables were expressed as counts and percentages.

Baseline characteristics were compared between SGLT2i and Non-SGLT2i groups using an independent samples *t*-test or Mann–Whitney U test for continuous variables, and χ^2^ test for categorical variables.

Longitudinal changes between T0 and T8 were evaluated using repeated-measures analysis of variance (ANOVA), with time as the within-subject factor and treatment group as the between-subject factor. Prior to analysis, normality of residuals and homogeneity of variances were assessed; sphericity was evaluated using Mauchly’s test. When assumptions were violated, the Greenhouse-Geisser correction was applied.

The primary outcomes were changes (delta) in PRAT and EAT thickness (ΔPRAT and ΔEAT). Associations between SGLT2i exposure and ΔPRAT/ΔEAT were assessed using linear regression models. Both crude and multivariable-adjusted models were constructed. Multivariable models were prespecified to adjust for age, sex, baseline eGFR, diabetes status, and concomitant GLP-1 receptor agonist therapy. Sensitivity analyses further included body mass index (BMI) and visceral fat area (VFA) to evaluate independence from overall and visceral adiposity. Additionally, sensitivity analyses were performed adjusting for longitudinal changes in BMI (ΔBMI) to account for the potential impact of weight loss over time. Covariates included in multivariable models were prespecified based on biological plausibility rather than data-driven selection.

Secondary exploratory analyses examined associations according to SGLT2i subtype (dapagliflozin vs. empagliflozin). Multicollinearity was assessed using variance inflation factors (VIF < 5 considered acceptable). Model fit was evaluated using R^2^ and adjusted R^2^.

Missing data were present in the control group and handled with a complete-case approach. Given the observational nature of the study and the relatively low proportion of missingness, no imputation procedures were performed; however, this approach may introduce bias and should be considered when interpreting the results.

All analyses were performed using JASP© version 0.19.3 (Amsterdam, The Netherlands). A two-sided *p*-value < 0.05 was considered statistically significant.

### 2.5. Ethical Aspects

The research was conducted following the Declaration of Helsinki as revised in 2013. Authorization was gathered from the Virgen del Consuelo Hospital (code: 23.70), as well as ethical approval from the Biomedical Research Ethics Committee at CEU Cardenal Herrera University (approval code: CEEI23/424). Informed consent was obtained from all patients before being included in the study.

## 3. Results

### 3.1. Baseline Characteristics

The sample was composed of 189 participants with CKD at different stages (1–4). Fifty (26.45%) of them met criteria for SGLT2i treatment (SGLT2i cohort), whereas the remaining 139 (73.54%) continued in the Non-SGLT2i cohort.

Baseline demographic and clinical characteristics of the sample are summarized in Table 1. Patients in the SGLT2i cohort were mainly composed of males (62%) and tended to be older (*p* < 0.049). As reasonably expected, they had a significantly higher CV load, characterized by a higher prevalence of hypertension (*p* < 0.001), T2DM (*p* < 0.001) and a significantly higher percentage of patients categorized as CKD grades 3–4 (*p* < 0.002) in relation to those in the Non-SGLT2i cohort. Consequently, the intake of medications such as beta-blockers, finerenone, metformin, DPP4i, insulin, statins, allopurinol and paricalcitol was significantly higher in those candidates for SGLT2i, with no significant differences in GLP-1RA usage (*p*~0.327).

### 3.2. Primary Endpoints

#### 3.2.1. Longitudinal Changes in Cardiorenal Adipose Depots

***Perirenal adipose tissue***

**Figure 1:**
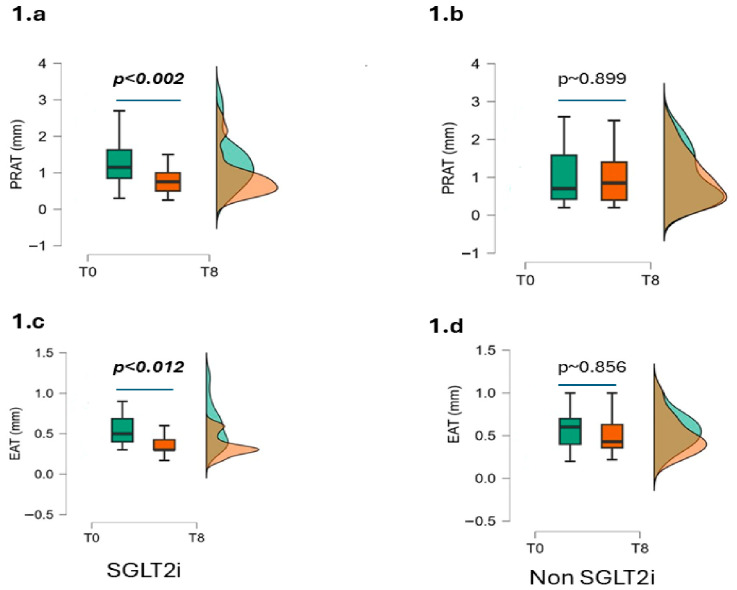
Time × treatment interaction on ectopic adipose depots. T0 represents baseline and T8 the 8-month follow-up. Panels (**a**) and (**b**) show PRAT changes in the SGLT2i and Non-SGLT2i groups, respectively, while panels (**c**) and (**d**) depict EAT changes. Analyses were performed using repeated-measures ANOVA. Changes in PRAT and EAT should be interpreted as longitudinal associations according to treatment group. Green color represents values at T0, whereas orange color depicts values at T8.

PRAT decreased from 1.28 ± 0.70 cm to 0.91 ± 0.60 cm after SGLT2i treatment, resulting into a ΔPRAT = −0.37 cm (*p* < 0.002), which was significantly higher than the difference in PRAT observed for the Non-SGLT2i cohort (ΔPRAT = −0.08 cm; *p*~0.899) (Table 2, Figure 1a,b), with an intergroup Cohen’s d for ΔPRAT =−0.63 (*p* < 0.018).

**Figure 2:**
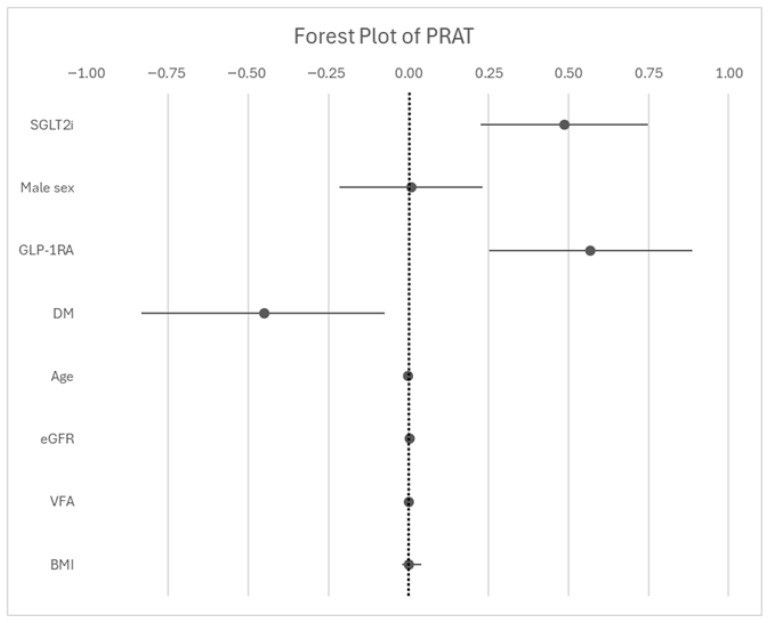
Multivariable linear regression analysis of factors associated with changes in perirenal adipose tissue (ΔPRAT). Regression coefficients (β) with 95% confidence intervals are shown. Negative β values indicate greater reductions in PRAT during follow-up. Results represent adjusted associations and do not imply causality. Abbreviations: SGLT2i= sodium–glucose cotransporter 2 inhibitors; GLP-1RA= glucagon-like peptide 1 receptor agonist; DM= diabetes mellitus; eGFR = estimated glomerular filtration rate; VFA = visceral fat area; BMI = body mass index.

In unadjusted linear regression analysis, SGLT2i treatment was significantly associated with ΔPRAT (β = 0.295, 95% CI 0.052 to 0.537, *p* < 0.018), explaining approximately 8% of the variance in ΔPRAT (R^2^ = 0.084). After adjustment for age, sex, baseline eGFR, diabetes mellitus status, and concomitant use of GLP-1RA, SGLT2i therapy remained independently associated with ΔPRAT (β = 0.447; 95% CI 0.211 to 0.682; *p* < 0.001). The adjusted model explained 37% of the variance in ΔPRAT (R^2^ = 0.371), with collinearity < 5 (all VIF values < 1.5). Further sensitivity analyses adjusting for BMI and VFA suggested that the association was not fully explained by overall or visceral adiposity (Figure 2).

***Epicardial adipose tissue***

A significant reduction in EAT was found after SGLT2i treatment for 8 months (from 0.57 ± 0.27 cm to 0.36 ± 0.14 cm, ΔEAT = −0.21, *p* < 0.012), whereas no significant change in EAT was observed in the Non-SGTL2i cohort (ΔEAT = −0.06, *p*~0.856) (Table 2, Figure 1c,d).

**Figure 3:**
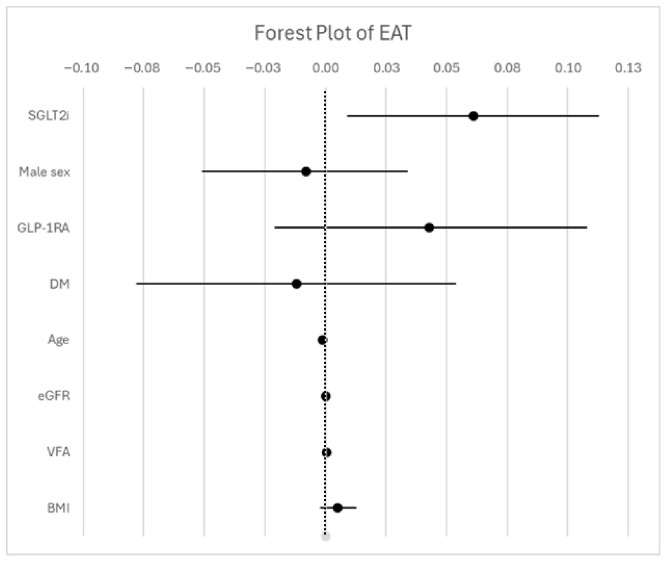
Multivariable linear regression analysis of factors associated with changes in epicardial adipose tissue (ΔEAT). Regression coefficients (β) with 95% confidence intervals are shown. Negative β values indicate greater reductions in EAT during follow-up. Results represent adjusted associations and should be interpreted within the limitations of an observational design. Abbreviations: SGLT2i= sodium–glucose cotransporter 2 inhibitors; GLP-1RA= glucagon-like peptide 1 receptor agonist; DM= diabetes mellitus; eGFR = estimated glomerular filtration rate; VFA = visceral fat area; BMI = body mass index.

Unadjusted linear regression analysis revealed a significant association between SGLT2i and ΔEAT (β = 0.060, 95% CI 0.014 to 0.106, *p* < 0.011, R^2^ = 0.033). This association remained statistically significant after multivariable adjustment, including GLP-1 receptor agonist use, diabetes status, sex, age, renal function, BMI and VFA (β = 0.061, 95% CI 0.009 to 0.113, *p* < 0.021). The adjusted model explained 5.3% of the variance in ΔEAT (R^2^ = 0.053), with collinearity values (VIF) < 5 (Figure 3).

#### 3.2.2. Longitudinal Renal, Metabolic, and Inflammatory Changes

During the 8-month follow-up, no significant differences were observed in either of the cohorts for most of the cardiometabolic, renal, inflammatory and body composition parameters (Table 2).

Anthropometric measures revealed non-significant improvements in BMI, body fat percent and VFA in both groups, whereas lipid and glycemic control remained stable over time. Renal function biomarkers were marked by considerable reductions in microalbuminuria and urinary albumin-to-creatinine ratio (ACR) after SGLT2i treatment (microalbuminuria: from 101.07 mg/L to 49.89 mg/L, *p*~0.409; and ACR: from 143.05 mg/g to 89.91 mg/g, *p*~0.191). Changes in inflammatory variables were characterized by non-significant reductions in leptin, IL-6 and TNF-α, most notably after SGLT2i administration.

### 3.3. Secondary Endpoints

#### Exploratory Analysis by SGLT2i Subtype

Twenty-six patients on the SGLT2i cohort (52%) were treated with empagliflozin, whereas 48% received dapagliflozin. To secondarily explore potential differences between agents, multivariable regression analyses for PRAT and EAT were repeated according to the SGLT2i subtype (Non-SGLT2i, dapagliflozin, empagliflozin).

In multivariable linear regression adjusted for GLP-1RA therapy, diabetes, sex, age, eGFR, BMI and VFA, an association between dapagliflozin and ΔPRAT was observed (R^2^ = 0.173; β = −0.252, 95% CI −0.373 to −0.132, *p* < 0.002), whereas the association between ΔPRAT and empagliflozin was a non-significant decrease (*p*~0.165). On the contrary, empagliflozin was significantly associated with ΔEAT in the multivariable linear regression model (R^2^ = 0.057; β = −0.072, 95% CI −0.137 to −0.007, *p* < 0.030), whereas a non-significant decrease association was observed between dapagliflozin and ΔEAT (*p*~0.248). These exploratory findings should be interpreted with caution, as the study was not powered for subtype comparisons and the observed differences may reflect random variation rather than true pharmacological heterogeneity.

## 4. Discussion

In this prospective and longitudinal cohort study of CKD patients, our results show for the first time that SGLT2i therapy was associated with significant reductions in both EAT and PRAT thickness. Importantly, these reductions remained independently associated with SGLT2i exposure after adjustment for GLP-1RA use, diabetes status, renal function, BMI, body composition and visceral adiposity. In a secondary analysis, independent associations between dapagliflozin and PRAT and between empagliflozin and EAT were found, suggesting a potential differential depot responsiveness according to SGLT2i subtype. Collectively, these findings are consistent with an association between SGLT2i exposure and remodeling across ectopic adipose depots; however, given the observational design, these results should be interpreted as associative and hypothesis-generating rather than indicative of causal or therapeutic effects.

SGLT2is are firmly established as a cardiorenal protective therapy across diabetic and non-diabetic populations [11,20]. However, the structural and tissue-level mechanisms underlying these benefits remain unknown. Increasing attention has been directed towards ectopic adipose depots as active mediators of organ injury rather than passive fat storage compartments [21,22]. In this context, our findings provide novel clinical evidence that SGLT2i therapy may influence adipose tissue biology at cardiac and renal compartments in CKD-affected populations.

EAT is a metabolically active visceral depot with direct paracrine and vasocrine effects on the myocardium [23,24]. Increased EAT has been associated with myocardial inflammation, fibrosis, and adverse cardiac remodeling [25,26]. The observed reduction in EAT thickness in our cohort aligns with prior studies reporting on SGLT2i-mediated modulation of local inflammatory signaling and adipocyte metabolism [27,28,29]. Of note, the magnitude of EAT reduction (Δ ≈ −0.21 cm) should be interpreted with caution, as the clinical implications of such changes remain incompletely defined. Notably, similar reductions in EAT have been reported in studies conducted in the general and type 2 diabetes populations, suggesting that this effect may not be restricted to CKD but rather represents a broader cardiometabolic phenomenon.

PRAT represents a particularly relevant interface yet underexplored in CKD. Given its vascular and neural connections with the renal cortex [9], perirenal fat may contribute to altered intrarenal hemodynamics, sympathetic activation, and pro-inflammatory signaling [30,31,32]. Our results show that PRAT thickness decreased significantly over follow-up in those patients receiving SGLT2is, with a clear time-by-treatment interaction. Although SGLT2is are known to induce modest weight loss, the observed associations remained consistent after adjustment for BMI and visceral adiposity. This suggests that depot-specific changes may not be solely driven by global adiposity reduction. Similar to EAT, the magnitude of PRAT reduction observed in this study (Δ ≈ −0.37 cm) is relatively modest, and its direct clinical significance remains uncertain, as no established thresholds currently define clinically meaningful changes in PRAT in CKD populations. However, residual confounding related to changes in body composition over time cannot be fully excluded and should be considered when interpreting these findings.

Our longitudinal biochemical results further support this interpretation. Stabilization of kidney function accompanied by a downward trend in inflammatory biomarkers occurred despite minimal changes in traditional metabolic parameters. These observations reinforce the hypothesis that the observed PRAT and EAT remodeling may not simply reflect generalized metabolic improvement but rather a preferential organ-specific adipose and inflammatory modulation.

An additional exploratory finding was the observation of potential depot-specific responses according to the SGLT2i subtype, with dapagliflozin and empagliflozin showing differential associations with PRAT and EAT, respectively. This sub-analysis should be taken with caution, as our sample group sizes were small and this study was not powered for a head-to-head comparison between the two associations. The observed differences between the SGLT2i subtypes may instead reflect random variation rather than true biological differences.

Our results align with previous literature in non-CKD populations, such as the open-label randomized controlled trial conducted on patients with T2DM and obesity [26], who demonstrated a significant reduction in PRAT thickness after the administration of combined dapagliflozin and metformin compared with only metformin. Other placebo-controlled clinical trials, such as the SIMPLE study and the EMPA-TROPISM trial, revealed significant reductions in EAT in T2DM and non-diabetic patients, respectively [33]. As commented, although the study was not powered for head-to-head comparisons, these findings raise the possibility that organ-specific adipose tissue responses may vary across molecules within the same pharmacological class. Larger adequately powered studies are required to determine whether these differences represent true biological heterogeneity or reflect variability related to sample size and baseline characteristics.

Adipose tissue improvements occurred despite this less favorable baseline phenotype, which may suggest a consistent association across different baseline risk profiles. Nevertheless, residual confounding inherent to observational designs cannot be excluded.

SGLT2i are also intrinsically linked to broader anti-atherosclerotic properties, including improved lipid metabolism, enhanced endothelial function, and plaque stability through fibrous cap thickening [12]. These systemic benefits are driven by reduced vascular inflammation and oxidative stress, which, alongside the modulation of pro-inflammatory adipokine secretion, could contribute to reducing cardiovascular risk and managing CKD progression [12].

Beyond systemic metabolic shifts, the coordinated remodeling of EAT and PRAT observed in our study may reflect underlying changes in adipose tissue biology; however, these interpretations remain speculative. While prior experimental and clinical studies have suggested potential effects of SGLT2i on mitochondrial function, oxidative stress, and adipocyte phenotype, no mechanistic or molecular data were collected in the present study [16]. Therefore, hypotheses related to mitochondrial reprogramming or adipose tissue browning should be considered as conceptual frameworks rather than demonstrated mechanisms within our dataset.

Several limitations should be considered. First, this was a single-center observational study with non-randomized treatment allocation. Second, the sample size was modest and the follow-up duration relatively short. In addition, patients receiving SGLT2i had a higher baseline cardiovascular and renal burden; while we adjusted for these factors, the possibility of residual confounding by indication remains a limitation. Third, adipose depots were assessed using ultrasound rather than volumetric imaging modalities, as ultrasound studies are easier to conduct and cheaper to reproduce compared to MRI studies. Additionally, ultrasound examinations are part of the standard clinical routine at our hospital. Furthermore, intra- and inter-observer variability were not systematically assessed, which may limit the reproducibility and precision of adipose tissue measurements. No histological or molecular characterization of tissue changes was performed. Importantly, this study was not designed to investigate mechanistic pathways, and the proposed biological interpretations should be considered hypothesis-generating rather than confirmatory. Finally, the inclusion of heterogeneous CKD stages and cardiometabolic profiles may introduce biological variability.

Regarding future investigations, several critical research gaps could be addressed to move beyond the correlational nature of our current data. While our findings highlight a significant association between SGLT2i use and adipose remodeling, future studies should prioritize the use of gold-standard volumetric imaging, such as MRI or CT, to confirm these ultrasound-based measurements with higher anatomical precision. Furthermore, there is a pressing need for multi-center randomized controlled trials that include larger, more diverse cohorts across varying stages of CKD to ensure the generalizability of these results. Mechanistically, prospective studies incorporating biopsy-based histological or transcriptomic analysis of EAT and PRAT are essential to determine whether SGLT2i induces a true “browning” of white adipose tissue or primarily acts by reducing local adipocyte hypertrophy and pro-inflammatory cytokine secretion. Additionally, head-to-head comparative trials between different SGLT2i molecules are warranted to determine if the differential responses observed in our sub-analysis represent a true class-specific biological heterogeneity or are simply a reflection of sample size variability. Such advancements would refine our understanding of ectopic fat as a therapeutic target in the cardiorenal syndrome framework.

## 5. Conclusions

The present study shows that SGLT2i therapy is independently associated with longitudinal reductions in epicardial and PRAT in patients with CKD stages 1–4. Given the observational design, these findings should be interpreted as exploratory associations. Further studies are needed to determine their clinical relevance and underlying mechanisms.

## Figures and Tables

**Table 1:** Initial (**T0**) comparisons of demographic and clinical data between patient cohorts (SGLT2i vs. Non-SGLT2i).

Variables	SGLT2i Cohort	Non-SGLT2i Cohort	*p*
**Nº of patients (%)**	50 (26.45)	139 (73.54)	-
**Sex: males (%)**	31 (62)	81 (41)	<0.012 ^§^
**Age (years): mean (SD)**	67.28 (13.70)	62.34 (15.61)	<0.049 ^†^
**SBP (mmHg): mean (SD)**	131 (16.95)	125.51 (11.96)	~0.147 ^†^
**DBP (mmHg): mean (SD)**	79.59 (8.97)	78.94 (11.37)	~0.832 ^†^
**Patients with hypertension: *n* (%)**	41 (82)	72 (51.80)	<0.001 ^§^
**Patients with T2DM: *n* (%)**	15 (30)	10 (7.20)	<0.001 ^§^
**Smoking: *n* (%)**	11 (22)	14 (10.07)	<0.022 ^§^
**CKD grades: *n* (%)** **-G1** **-G2** **-G3-G4**	5 (10) 14 (28) 31 (62)	33 (23.74) 59 (42.44) 47 (33.82)	<0.002 ^§^ <0.002 ^§^ <0.002 ^§^
**CVD load: *n* (%)** **-Myocardial infarction** **-Stroke** **-Peripheral vascular disease**	12 (24) 6 (12) 7 (14)	10 (7.19) 9 (6.47) 6 (4.31)	<0.001 ^§^ ~0.215 ^§^ <0.020 ^§^
**ACE inhibitors: *n* (%)**	11 (22)	17 (12.23)	~0.095 ^§^
**ARBs: *n* (%)**	27 (54)	45 (32.37)	<0.007 ^§^
**CCBs: *n* (%)**	13 (26)	26 (18.70)	~0.274 ^§^
**Diuretics: *n* (%)**	19 (38)	37 (26.62)	~0.131 ^§^
**Beta-blockers: *n* (%)**	16 (32)	23 (16.54)	<0.017 ^§^
**MRAs: *n* (%)**	14 (28)	19 (13.67)	<0.047 ^§^
**Finerenone (ns-MRAs): *n* (%)**	5 (10)	3 (2.15)	<0.018 ^§^
**Metformin: *n* (%)**	13 (26)	10 (7.19)	<0.010 ^§^
**DPP4i: *n* (%)**	8 (16)	6 (4.31)	<0.004 ^§^
**Insulin: *n* (%)**	6 (12)	1 (0.72)	<0.001 ^§^
**GLP-1RA: *n* (%)**	6 (12)	25 (17.98)	~0.327 ^§^
**Statins: *n* (%)**	35 (70)	56 (40.29)	<0.001 ^§^
**Fibrates: *n* (%)**	3 (6)	3 (2.16)	~0.194 ^§^
**Allopurinol: *n* (%)**	14 (28)	17 (12.23)	<0.010 ^§^
**Vitamin D and Calcitriol: *n* (%)**	7 (14)	12 (8.63)	~0.095 ^§^
**Paricalcitol: *n* (%)**	5 (10)	4 (2.87)	<0.043 ^§^

Statistical comparisons performed through: ^§^ Chi-square test of independence; ^†^ Independent samples Student’s *t*-test. Abbreviations: SGLT2i = sodium-glucose cotransporter 2 inhibitors; % = percentage; SD = standard deviation; SBP = systolic blood pressure; DBP= diastolic blood pressure: IQR = interquartile range; BMI = body mass index; n = number of patients; T2DM= type 2 diabetes mellitus; ADPKD = autosomal dominant polycystic kidney disease; CKD = chronic kidney disease; G1 = grade 1 of chronic kidney disease; G2 = grade 2 of chronic kidney disease; G3a= grade 3a of chronic kidney disease; G3b= grade 3b of chronic kidney disease; G4 = grade 4 of chronic kidney disease; CVD = cardiovascular disease; ACE = angiotensin-converting enzyme; ARBs= angiotensin receptor blockers; CCBs = calcium channel blockers; ns-MRAs= nonsteroidal mineralocorticoid receptor antagonists; DPP4i = dipeptidyl peptidase 4 inhibitors; GLP-1RA= glucagon-like peptide 1 receptor agonist. Alt text: table showing the baseline clinical information of our participants. Patients receiving SGLT2 inhibitors were older, more frequently male, and had a higher cardiometabolic and cardiovascular disease burden, including more advanced CKD stages, compared with the non-SGLT2i group. Accordingly, the use of cardiometabolic therapies was more frequent, while blood pressure values were similar between groups.

**Table 2:** Changes in cardiometabolic, inflammatory, renal and adipose tissue parameters with time as the within-subject factor (T0 vs. T8) and treatment as the between-subject factor (SGLT2i vs. Non SGLT2i).

Variables	SGLT2i			Non SGLT2i		
	T0	T8	*p*	T0	T8	*p*
**BMI (kg/m^2^): mean (SD)**	25.20 (3.67)	22.60 (3.75)	~0.743	28.61 (4)	26.75 (4.03)	~0.892
**Body Fat Percent (%): mean (SD)**	46.20 (7.90)	40.90 (9.38)	~0.900	36.86 (7.82)	36.34 (6.25)	~0.900
**Muscle Mass (kg): mean (SD)**	48.90 (11.14)	47.42(10.93)	~0.900	46.36 (10.29)	44.73 (10.32)	~0.627
**Visceral Fat Area (cm^2^): mean (SD)**	76.30 (42.77)	63.70 (37.46)	~0.900	137.48 (58.11)	101.79 (31.73)	~0.192
**Total Body Water (L): mean (SD)**	38.51 (8.66)	37.28 (8.72)	~0.900	36.26 (7.81)	35.12 (7.89)	~0.688
**Hematocrit (%): mean (SD)**	44.88 (4.81)	45.20 (4.30)	~0.767	43.07 (4.84)	43.33 (4.35)	~0.767
**Glucose (mg/dL): mean (SD)**	111.62 (23.37)	108.29 (22.06)	~0.261	101.93 (19.56)	97.01 (10.94)	~0.161
**Urea (mg/dL): mean (SD)**	52.40 (22.41)	50.68 (20.46)	~0.491	43.33 (16.86)	46.76 (16.11)	~0.620
**Creatinine (mg/dL): mean (SD)**	1.27 (0.36)	1.26 (0.37)	~0.747	1.03 (0.32)	1.01 (0.30)	~0.743
**eGFR (ml/min/1.73 m^2^): mean (SD)**	58.55 (23.05)	58.98 (19.84)	~0.900	67.82 (22.18)	70.60 (22.96)	~0.247
**Uric acid (mg/dL): mean (SD)**	5.83 (1.94)	5.79 (1.65)	~0.9000	5.98 (1.60)	5.70 (1.46)	~0.646
**Albumin (g/L): median (IQR)**	4.55 (0.40)	4.60 (0.40)	~0.900	4.50 (0.40)	4.50 (0.20)	~0.900
**Calcium (mg/dL): mean (SD)**	9.68 (0.44)	9.70 (0.39)	~0.900	9.71 (0.53)	9.68 (0.45)	~0.900
**Phosphate (mg/dL): mean (SD)**	3.78 (0.53)	3.80 (0.57)	~0.900	3.45 (0.57)	3.66 (0.59)	~0.066
**LDL-cholesterol (mg/dL): mean (SD)**	84.46 (31.58)	79.15 (30.78)	~0.518	110.91 (38.98)	105.75 (31.07)	~0.518
**Triglycerides (mg/dL): mean (SD)**	126.30 (57.56)	125.80 (65.35)	~0.931	106.20 (43.16)	102.10 (40.39)	~0.160
**Glycated hemoglobin (%): median (IQR)**	6.13 (0.77)	6.16 (0.81)	~0.761	5.73 (0.44)	5.78 (0.55)	~0.761
**iPTH (pg/mL): mean (SD)**	57.75 (20.65)	63.76 (20.11)	0.900	52.03 (23.94)	52.82 (25.33)	~0.900
**Ferritin (ng/mL): mean (SD)**	196.70 (140.30)	180.20 (131.63)	~0.701	131.10 (72.91)	129.50 (70.87)	~0.914
**Leptin (ng/mL): mean (SD)**	25.83 (25.86)	22.15 (19.08)	~0.337	44.33 (31.45)	38.63 (26.49)	~0.239
**IL-6 (pg/mL): mean (SD)**	7.88 (14.99)	3.71 (2.36)	~0.901	3.61 (1.65)	3.44 (1.71)	~0.900
**CRP (mg/dL): mean (SD)**	0.18 (0.16)	0.28 (0.40)	~0.868	0.60 (1.67)	0.47 (0.86)	~0.689
**TNF-α (ng/mL): mean (SD)**	11.70 (8.29)	9.05 (3.99)	~0.548	8.36 (2.91)	7.82 (1.81)	~0.900
**MA (mg/L): mean (SD)**	101.07 (259.11)	49.89 (84.11)	~0.409	15.38 (41.22)	21.10 (56.65)	~0.823
**Urinary ACR (mg/g): mean (SD)**	143.05 (284.54)	89.91 (181.92)	~0.191	15.74 (28.68)	24.31 (61.27)	~0.730
**Urinary PCR (mg/g)**	0.34 (0.43)	0.34 (0.39)	~0.900	0.09 (0.04)	0.10 (0.07)	~0.900
**PRAT (cm): mean (SD)**	1.28 (0.70)	0.91 (0.61)	<0.002	1.02 (0.69)	0.94 (0.63)	~0.899
**EAT (cm): mean (SD)**	0.57 (0.27)	0.36 (0.14)	<0.012	0.56 (0.20)	0.50 (0.22)	~0.856

Statistical comparisons were performed with repeated-measures analysis of variance (ANOVA) and Bonferroni post hoc analysis. Abbreviations: GLP-1RA= glucagon-like peptide 1 receptor agonist; SD = standard deviation; eGFR = estimated glomerular filtration rate; LDL-cholesterol = low-density lipoprotein cholesterol; % = percentage; IQR = interquartile range; iPTH = intact parathyroid hormone; TNF-α= tumor necrosis factor-alpha; IL-6 = interleukin-6; CRP = C-reactive protein; MA = microalbuminuria; ACR = albumin-to-creatinine ratio; PCR = protein-to-creatinine ratio; PRAT = perirenal adipose tissue; EAT = epicardial adipose tissue. Alt text: table showing the changes in anthropometric, analytical and imaging data in both groups of participants, those under SGLT2i and those without this treatment. Over the 8-month follow-up, most cardiometabolic, renal and inflammatory parameters did not show statistically significant changes, whereas significant reductions were observed in PRAT and EAT in the SGLT2i group, consistent with the primary study outcomes. Primary outcomes of interest (PRAT and EAT) are in bold to emphasize the main study objectives. Other variables are shown for completeness and should be interpreted as exploratory.

## Data Availability

The datasets generated and analyzed during the current study are available from the corresponding author upon reasonable request. Due to ethical restrictions involving human participants, raw data is not publicly available in order to protect participant privacy and confidentiality.
